# Prevalence of SARS-CoV-2 antibodies in hemodialysis patients in Senegal: a multicenter cross-sectional study

**DOI:** 10.1186/s12882-021-02582-w

**Published:** 2021-11-17

**Authors:** Sidy Mohamed Seck, Moustapha Mbow, Yaya Kane, Mouhamadou Moustapha Cisse, Gnagna Faye, Adama Kama, Moussa Sarr, Pamela Nitcheu, Mohamed Dahaba, Ibrahima Mbemba Diallo, Mame Selly Diawara, Lotingo Nehemie Motoula Latou, Yacine Dia, Souleymane Mboup

**Affiliations:** 1 Department of Nephrology/Internal Medicine, Faculty of Health Sciences, University Gaston Berger of Saint-Louis, Route de Ngallele, BP 234, Sanar, Saint-Louis, Senegal; 2 Dialysis Center, Military Hospital of Ouakam, Dakar, Senegal; 3 IRL-ESS-3189, Faculty of Medicine, University Cheikh Anta Diop, Dakar, Senegal; 4 University Cheikh Anta Diop, Dakar, Senegal; 5 University Assane Seck, Ziguinchor, Senegal; 6 University of Thies, Thiès, Senegal; 7 HOGIP, Dakar, Senegal; 8 CHR EIN, Kaolack, Senegal; 9 CHR Louga, Louga, Senegal; 10 CHR Tamba, Tambacounda, Senegal; 11 CHN Fawzeyni, Touba, Senegal; 12 IRESSEF Diamniadio, Dakar, Senegal

**Keywords:** SARS-CoV-2, COVID-19, Seroprevalence, Hemodialysis, Senegal

## Abstract

**Background:**

Hemodialysis patients are among high-risk groups for COVID-19. Africa is the continent with the lowest number of cases in the general population but we have little information about the disease burden in dialysis patients.

**Objectives:**

This study aimed to describe the seroprevalence of SARS-CoV-2 antibodies in the hemodialysis population of Senegal.

**Patients and methods:**

We conducted a multicenter cross-sectional survey, between June and September 2020 involving 10 public dialysis units randomly selected in eight regions of Senegal. After seeking their consent, we included 303 patients aged ≥ 18 years and hemodialysis for ≥ 3 months. Clinical symptoms and biological parameters were collected from medical records. Patients’ blood samples were tested with Abbott SARS-CoV-2 Ig G assay using an Architect system. Statistical tests were performed with STATA 12.0.

**Results:**

Seroprevalence of SARS-CoV-2 antibodies was 21.1% (95% CI = 16.7–26.1%). We noticed a wide variability in SARS-CoV-2 seroprevalence between regions ranging from 5.6 to 51.7%. Among the 38 patients who underwent nasal swab testing, only six had a PCR-confirmed infection and all of them did seroconvert. Suggestive clinical symptoms were reported by 28.1% of seropositive patients and the majority of them presented asymptomatic disease. After multivariate analysis, a previous contact with a confirmed case and living in a high population density region were associated with the presence of SARS-CoV-2 antibodies.

**Conclusion:**

This study presents to our knowledge the first seroprevalence data in African hemodialysis patients. Compared to data from other continents, we found a higher proportion of patients with SARS-CoV-2 antibodies but a lower lethality rate.

## Introduction

The severe acute respiratory syndrome coronavirus 2 (SARS-CoV-2) has become a global health issue since its description in December 2019 in China [1]. Pandemic was declared by WHO at the end of January 2020 as the disease spread to all continents and imposed perturbations in healthcare, socio-economic and political systems [2, 3]. Patients with end-stage renal disease receiving dialysis treatment are very exposed to the SARS-CoV-2 during their frequent visits to healthcare facilities and immune dysfunction induced by uremia. Moreover, available data identified them as among the highest risk groups for severe cases and death upon contracting COVID-19 [2]. Since the pandemic declaration, various strategies to reduce the risk of COVID-19 infection in patients receiving in-center hemodialysis have been rapidly implemented in many countries [3]. In Senegal, such measures were implemented in all the 20 public dialysis centers receiving a cohort of 1100 patients with about 380 annual incident patients. Despite these measures, high numbers of COVID-19 cases and related death have been continuously reported in the world. For unclear reasons, the African continent was less hit by the pandemic with lower incident cases and death in the general population [4] and patients with ESRD [5]. Among the most likely hypotheses to explain this African exception is the possible cross-immunity with other common pathogens, younger mean age, lower life expectancy, smaller pool of old people surviving and living with chronic non-communicable diseases [4]. However, given the majority of asymptomatic cases, the real burden of the COVID-19 pandemic might be underestimated in absence of a massive testing strategy (PCR or serology) in the population [6]. To our knowledge, seroprevalence data in African hemodialysis patients has not been reported yet. This study investigated the prevalence of SARS-CoV-2 serologic markers and their associated factors in a nationally representative cohort of hemodialysis patients in Senegal.

## Patients and methods

We performed a cross-sectional multicenter study between June and September 2020 involving 10 dialysis units randomly selected in eight regions of Senegal. Patients aged ≥ 18 years, on chronic hemodialysis for at least 3 months were included. Patients who did not give their consent and those with a diagnosis of acute kidney injury were excluded. For each patient, we collected past clinical symptoms during 3 months and dialysis parameters from medical records. We also collected information about previous positive PCR tests in patients who were presented symptoms or were in contact with a confirmed COVID-19 case. Following the national COVID-19 management protocol, only patients with suspected clinical symptoms were proposed to do nasal swab tests for RT-PCR. Biological parameters were obtained from blood samples collected before one mid-week dialysis session during routine laboratory check-ups of patients.

### SARS-CoV-2 antibody detection

Patients’ samples were tested with the Healgen IgM/IgG One Step Rapid Test which measures separately the antibodies IgM and IgG. The test was run according to the manufacturer instructions. Into the test window, we added 5μl of serum and three drops of buffer provided in the kit. Then, results were read after 10 min (max 15 min) and only tests in which the control line changed its color were considered as valid. If a line was observed for IgM and/or IgG, the test was considered positive. The intensity of the color was not judged. According to the manufacturer, the specificity evaluated on PCR-negative samples and was 100% for both IgM and IgG, while the sensitivity evaluated on COVID-19 cases was calculated at 87.9% for IgM and 97.2% for IgG. Li et al. have shown an overall testing sensitivity of 88.7 and 90.6% specificity [7]. We have also evaluated the testing kit and found a sensitivity of 76.26% and specificity of 98.48% for IgM and a sensitivity of 83.38% and specificity of 96.96% for IgG. In recovery patients, the IgG sensitivity was 98.1% and the specificity 96,96% (unpublished data).

The study protocol was submitted to and approved by the “Ethical Committee of Faculty of Medicine, Pharmacy and Odontostomatology” at University Cheikh Anta Diop, (Dakar, Senegal).

Statistical analyses were performed using STATA 12.0 and R. Continuous variables were described as mean (standard deviation) or median (interquartile range). Normally distributed variables were compared with t-test, and nonparametric data were compared with the Mann-Whitney test. Categorical variables were presented as percent and Fisher exact tests or chi-squared tests were used for proportional assessments. For all statistical tests, we accepted two-sided level of significance was set at p≤ 0.05.

## Results

**Fig. 1:**
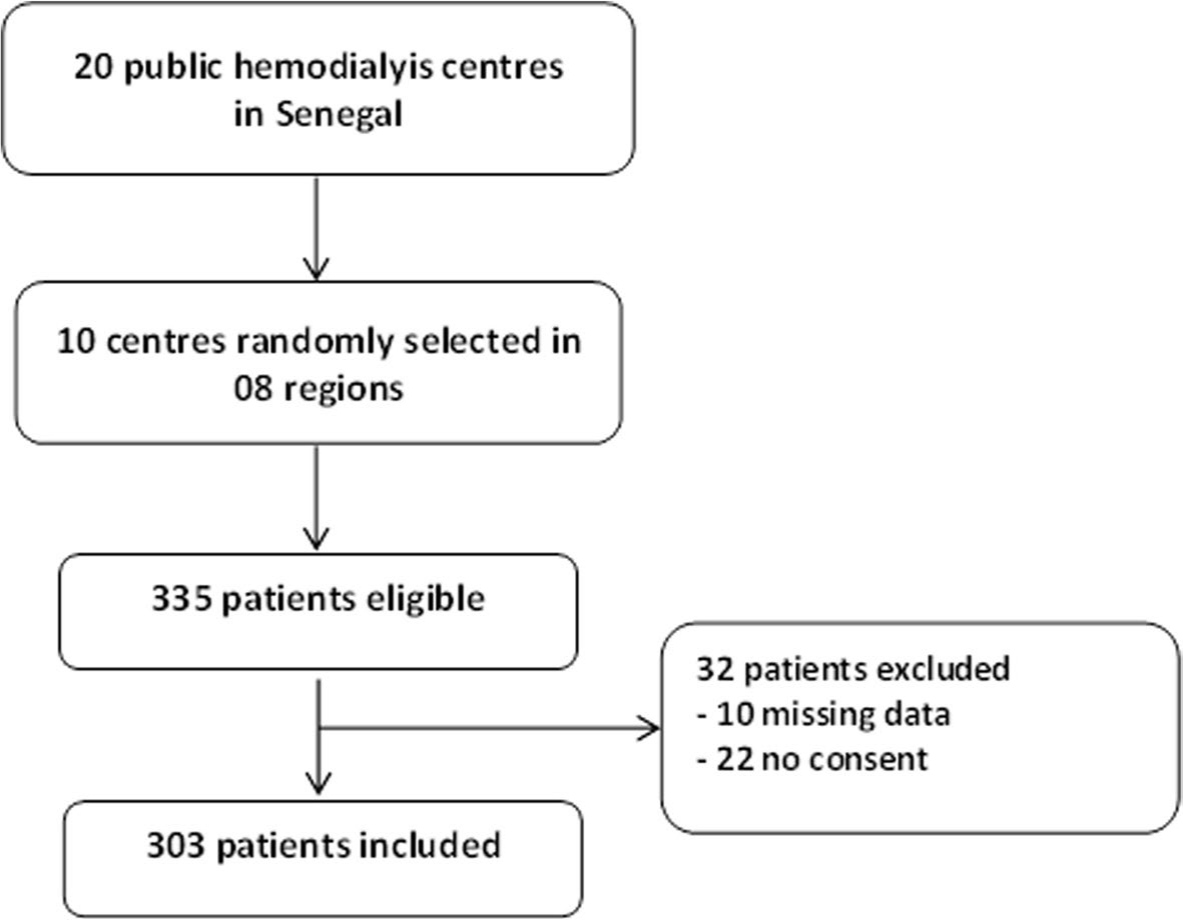
Patient flow

Out of 335 eligible hemodialysis patients, 303 were included in the study and had serologic testing for SARS-CoV-2 (Fig. 1). Sixty-four patients presented antibodies against the virus (seroprevalence of 21.1% with a 95% CI between 16.7 and 26.1%). About 95.3% of them had IgG antibodies. Table 1 presents socio-demographical characteristics and dialysis parameters of patients.

**Table 1:** Socio-demographical characteristics and dialysis parameters of patients

Variables	Seropositive patients (*n* = 64)	Seronegative patients (*n* = 239)	*P*-value
Age (years)	40.8 (16.7)	42.2 (18.1)	0.146
Age groups			0.652
< 40 years	30 (46.9%)	143 (59.8%)	
40–65 years	25 (39.1%)	69 (28.9%)	
> 65 years	9 (14.0%)	27 (11.3%)	
Gender			0.609
Male	38 (59.4%)	131 (54.8%)	
Female	26 (40.6%)	108 (45.2%)	
Educational level			0.808
Primary	23 (35.9%)	82 (34.3%)	
Secondary	14 (21.9 %)	61 (25.5 %)	
University	8 (12.5%)	28 (11.7%)	
Illiterate	19 (29.7 %)	68 (28.5 %)	
Dialysis vintage (months)^a^	26 (17)	29 (19)	0.533
Dry weight (kg)^a^	52.7 (34.3)	54.1 (22.5)	0.132
Body mass index (kg/m^2^)^b^	24.5 (13.1)	22.9 (17.4)	0.209
Dialysis frequency			0.002
< 3 sessions /week	25 (39.1%)	47 (19.7%)	
≥ 3 sessions /week	39 (60.9%)	192 (80.3%)	
Previous COVID19 PCR test			0.013
Positive	6 (33.3%)	0 (0%)	
Negative	12 (66.7%)	20 (100%)	

^a^median (interquartile range); ^b^mean (standard deviation)

**Fig. 2:**
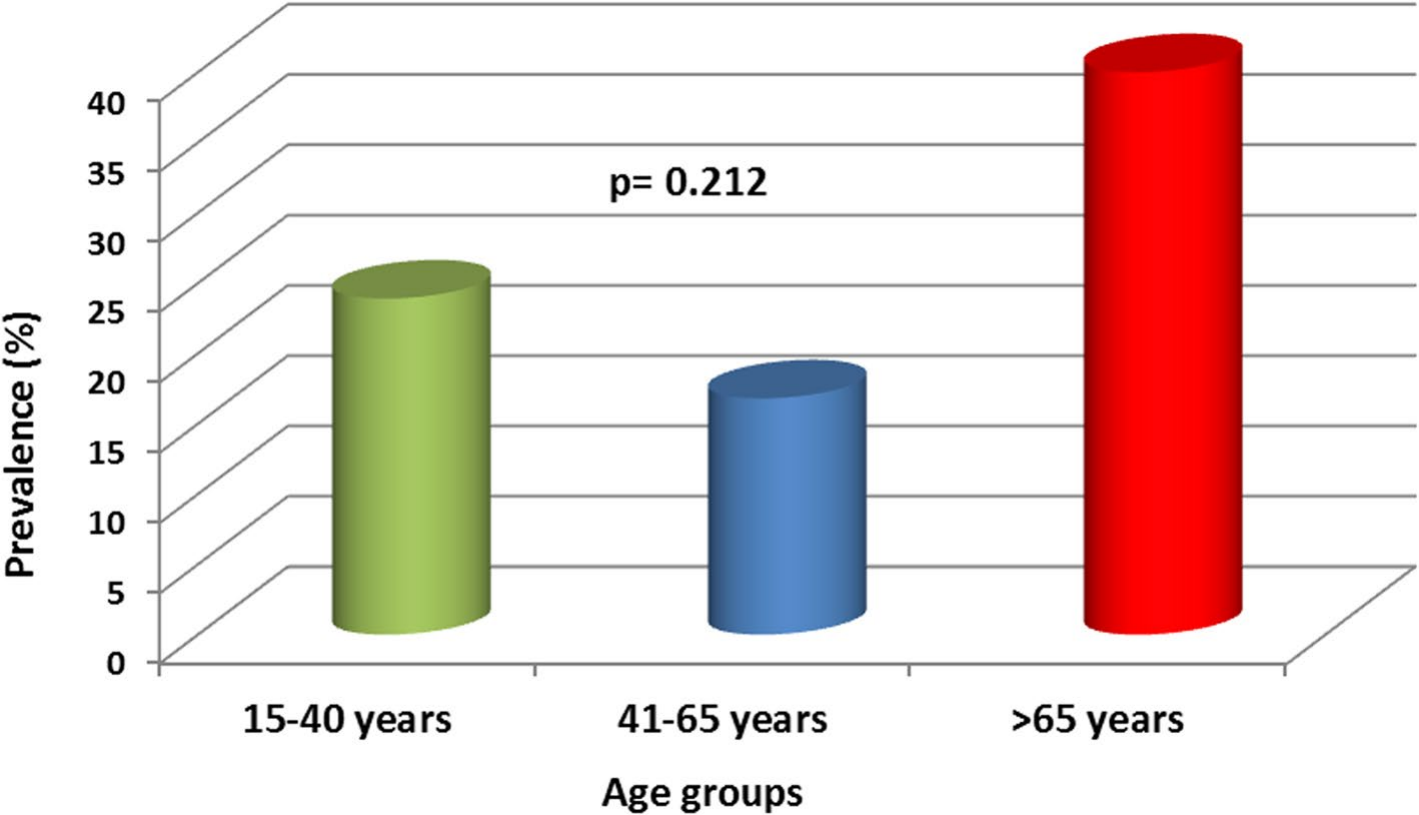
Prevalence of SARS-CoV2 antibodies by age groups

Seroprevalence of SARS-CoV-2 was comparable in men (24.3%) compared to women (17.2%) (*p* = 0.13) and tended to be higher in patients older than 65 years even this was not statistically significant (Fig. 2).

**Fig. 3:**
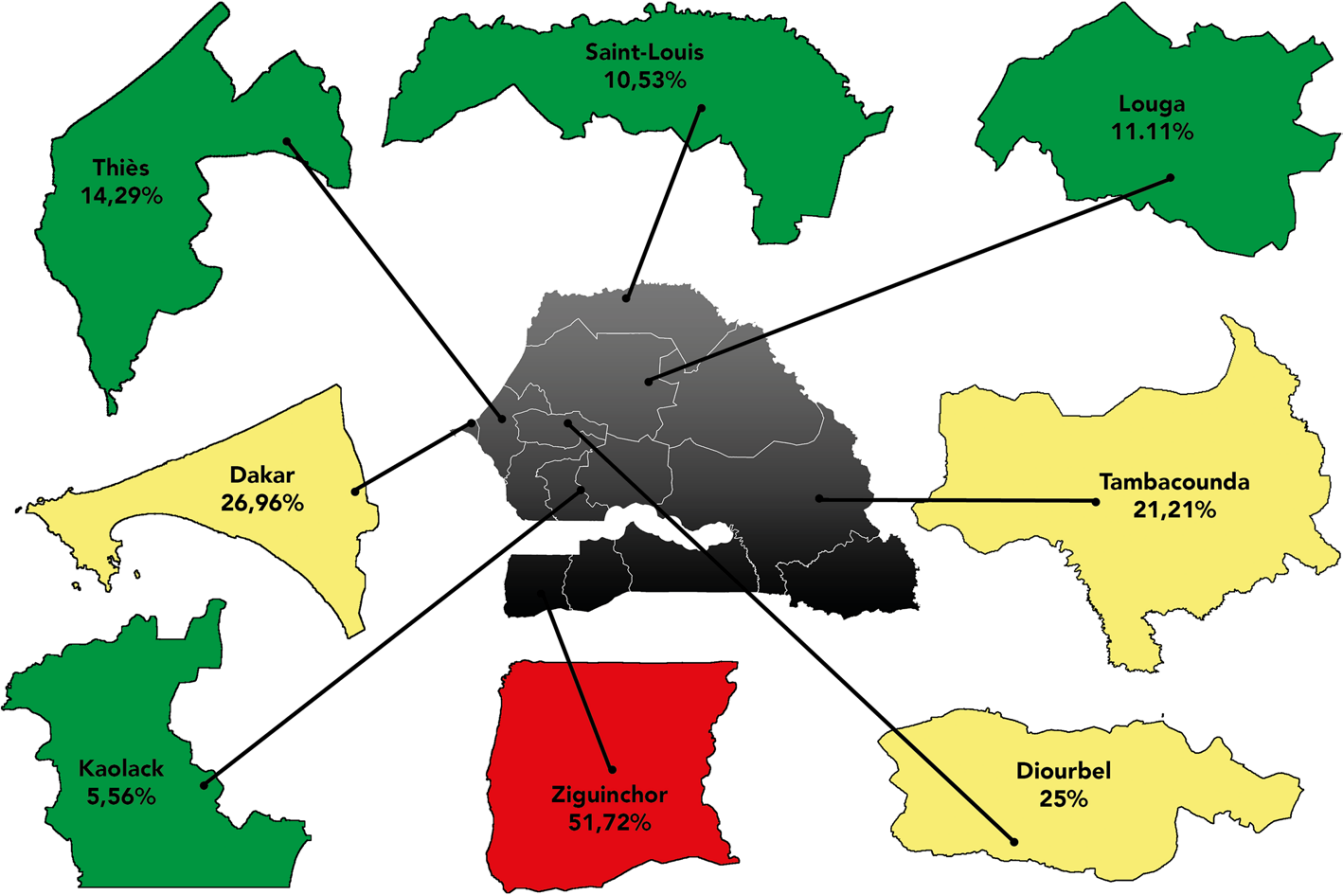
Geographical distribution of SARS-CoV2 antibodies

Also, we found a remarkable variability in SARS-CoV-2 seroprevalence between regions ranging from 5.6 to 51.7%. Patients from dialysis centers in the three regions of Dakar, Diourbel, and Ziguinchor presented a higher rate of seroconversion compared to those who lived in the rest of the country (Fig. 3).

Among the 38 patients (13 in Tambacounda, 10 in Dakar, 9 in Ziguinchor , 3 in Louga and 3 in Saint-Louis) who underwent nasal swab testing in the past 3 months, only six patients had a PCR-confirmed infection and all of them did seroconvert. Of the 64 patients with SARS-CoV-2 antibodies, only 18 (28.1%) presented suggestive clinical symptoms. The majority of them had an asymptomatic disease and did not have any PCR testing. Patients with positive SARS-CoV-2 serology experienced significantly more respiratory symptoms and fever than those who were seronegative. However, severe anemia and lymphopenia were not more frequent among COVID-19 patients (see Table 2). Serology tests were repeated 3 months later in 25 patients with SARS-CoV-2 antibodies and IgG persisted in 20 of them without evidence of new clinical symptoms.

**Table 2:** Clinical symptoms and COVID19 case contact history in patients

Characteristics	Seropositive patients (*n* = 64)	Seronegative patients (*n* = 239)	*P*-value
**Clinical symptoms**			
Fever	28 (43.7%)	46 (19.2%)	0.014
Respiratory symptoms	35 (54.7%)	41 (17.1%)	0.001
Digestive symptoms	19 (29.7%)	49 (20.5%)	0.389
**History of COVID19 case contact**			< 0.001
Yes	43 (67.2%)	83 (34.7%)	
No	21 (32.8%)	156 (65.3%)	

At univariate analysis, a positive COVID-19 serologic test was associated with clinical symptoms, known contact with a confirmed COVID-19 case, and living area.

However, after multivariate logistic regression analysis, only previous contact with a confirmed case, clinical respiratory symptoms and living a high population density region were associated with the presence of SARS-CoV-2 antibodies (see Table 3).

**Table 3:** Factors associated with SARS-Cov-2 seropositivity

Parameters	Univariate analysis		Multivariate analysis	
	OR [95% CI]	*p*	OR [95% CI]	*p*
Age	0.86 [0.53–01.42]	0.57	1.01 [0.57–01.77]	0.30
Gender	1.54 [0.87–02.73]	0.13	1.64 [0.86–03.12]	0.13
Education level	0.76 [0.39–04.68]	0.42	0.55 [0.34–06.24]	0.54
Region density	3.33 [1.86–05.97]	<0.01	4.13 [2.09–08.17]	< 0.01
Dialysis vintage	0.99 [0.97–01.01]	0.30	0.99 [0.98–01.02]	0.90
Contact case	7.90 [1.41–44.15]	0.02	13.66 [2.14–87.35]	0.01
Frequency of dialysis	0.90 [0.49–01.63]	0.72	0.91 [0.46–01.81]	0.80
Respiratory symptoms	5.84 [2.75–12.43]	<0.01	4.02 [2.87–17.16]	< 0.01
Fever	3.22 [1.34–07.73]	0.01	2.58 [0.92–07.26]	0.07
Diarrhea	1.60 [0.54–04.72]	0.39	0.93 [0.25–03.40]	0.91

Among the infected patients, six (1.9%) were admitted to intensive care units and three deaths due to COVID-19 were recorded during the study period.

## Discussion

In this nationwide survey of COVID-19 seroprevalence among hemodialysis patients in Senegal, we found that more than one patient in five presented specific antibodies by end of October 2020. Data from previous studies in hemodialysis cohorts reported variable but lower seroprevalences with 2.2% in Wuhan, China [8], 3.3% in France [9], 10.7% in the United Kingdom [10], 14.6% in Brescia, Italy [11], and from 3.5 to 27.2% in the USA [12]. In a recent review by Kooman JP, et al. the incidence of COVID-19 confirmed by RT-PCR among dialysis patients varied between 0.03 and 19.6% [3].

Unexpectedly our patients aged > 65 years showed the highest seroprevalence contrasting with publications where elders in dialysis showed a lower exposition to SARS-CoV-2 but a higher lethality rate [9, 12–14]. The age structure of the dialysis population in Africa is very different from developed countries where the majority of dialysis patients are over 65 years old [9–11].

The existence of the previous contact with a COVID-19 case and population density were the strongest factors associated with a positive serology among hemodialysis patients. Regional disparities observed within the country have been also noticed in previous nationwide surveys [9, 12]. They could be explained by the intensity of virus circulation and promiscuity. In the present survey, regions hosting two-thirds of infected patients were the most densely populated. In these settings, patients used to take crowded public transports to come to dialysis centers. The same hypothesis was evoked by Anand et al in a US nationwide comparative study which found that residents of neighborhoods with high population density presented a ten times higher risk of seropositivity compared with residents of lowest density areas [12]. Another US study showed that seroprevalence in the general and hemodialysis population are similar [15]. Since the early pandemic, epidemiological studies suggested that SARS-CoV-2 basic reproduction number (R0) increases linearly with population density [16] and this was why clusters were observed in crowded and confined spaces, mass gatherings, and populous metropolitan areas [17, 18]. More recently, a study by Cherif A et al, demonstrated that the COVID19 reproductive rate R(t) was comparable between dialysis and general population [19].

In contrast to the remarkable differences noted between Africa and the rest of the world regarding the extent of COVID-19 in the general population, we found among the hemodialysis subgroup a proportion of patients infected with SARS-CoV-2 which was comparable to data reported in other continents. Some authors suggested that the lower burden of COVID-19 in African countries could be explained by a majority of asymptomatic cases that were not diagnosed [20]. COVID-19 in our hemodialysis patients presented as an asymptomatic disease in 71.9% of cases. Similar to our findings, fever, cough, dyspnea, and fatigue are the most common symptoms described in patients with COVID-19 [3, 21] but digestive or cerebral manifestations can be more prominent in some cases [22].

Many studies using either PCR or serology tests had pointed out the predominance of asymptomatic forms in hemodialysis patients [12, 23] and underlined the paramount importance of preventive measures for all patients and staff inside dialysis centers. Testing strategies focusing on symptomatic cases only are known to underestimate the burden of the COVID-19 as it was demonstrated in the US population during the first wave of the pandemic that fewer than 10% of adults developed antibodies against SARS-CoV-2, and fewer than 10% of those were diagnosed [12]. Furthermore, African populations might develop a slightly different immune system response against the virus modulated by the multiple interactions with environmental factors and pathogens [24, 25].

The low number of deaths among Senegalese hemodialysis patients is confirming the relatively fewer deaths reported in African populations since the first pandemic wave [4, 20]. However, data suggest a more deadly second wave with a 40% increase in deaths in the continent since January 2021 [26]. Studies in other continents showed a mortality rate between 20 and 32.8% in hemodialysis patients with COVID-19 [26–28]. The younger age in our cohort was the main explanation for the lower number of deaths despite a higher incidence of COVID-19 cases.

In absence of seroprevalence data in the Senegalese general population, our findings in this representative nationwide hemodialysis cohort could serve as a good proxy to guide decisions of health authorities in the elaboration of an evidence-based strategy against COVID-19. Hemodialysis patients represent a good sentinel population in which to study the evolution of the current pandemic. Because we performed a serological test using blood samples collected as part of routine medical care, our results might not be influenced by possible selection bias compared to other previous studies that used nasal swab RT-PCR tests usually reserved for symptomatic patients.

Thus, these data in hemodialysis patients present a great epidemiological interest because it can be linked to community-level data to enable evaluation and quantification of differences in SARS-CoV-2 prevalence across socio-demographic and geographical strata, and thus develop efficient strategies targeting the highest-risk groups and areas.

Recent immunological studies demonstrated that patients with mild COVID-19 forms might develop multipotent SARS-CoV-2-specific immune memory response and IgG antibodies against spike protein could persist after recovery at least for 3 to 6 months [28, 29]. By demonstrating that many hemodialysis patients had already evidence of exposure and developed an immune response to SARS-CoV-2, this study opens prospects for future vaccination strategies in this specific group. The rationale of mass vaccination without excluding recovered patients with enough SARS-CoV-2 Ig G is yet to be demonstrated and benefits should be balanced with possible side effects and costs particularly in hemodialysis patients [30].

## Limits

Although we used a large and representative sample of dialysis patients in Senegal, our study presents some limits. First, the antibodies IgM and IgG are detected day 3–7 days after a person is infected by SARS-CoV-2 which suggests the serology test used may miss early infected people. Secondly, the retrospective collection of clinical symptoms and routine biological analysis could induce some recall bias when information was not written in the patient medical record. Also, we did not have some socio-demographical parameters like employment status, income level, living space, household size, and type of transportation from house to the dialysis center that could influence patient’s risk of contamination. However, the current results in this sub-group of dialysis patients might not reflect the true estimates in the general population. These results probably underestimate overall seroprevalence because dialysis patients usually face restrictions in their socio-professional activities due to their condition and maybe less in contact with the general population [31, 32]. Furthermore, data from hepatitis B post-immunization studies had demonstrated that compared to the general population, patients receiving dialysis might have a weaker immune response to viral antigen and thus be less likely to develop protective antibodies [33].

Finally, the early release and implementation of national guidelines for prevention and control of COVID-19 cases in dialysis facilities might have reduced the risk of in-center infections among patients and health workers.

## Conclusion

This study showed that despite a high seroprevalence of SARS-CoV-2 antibodies in Senegalese hemodialysis patients the majority of them presented with asymptomatic disease. Some variations were noticed between age groups and geographical areas. Also, COVID-19 was associated with a low hospitalization and fatality rate in hemodialysis patients. These findings could help inform detection and management strategies that can gain effectiveness by combining both nasal swab RT-PCR and antibody screening and by sensitizing more dialysis patients and their neighborhoods on the COVID-19 preventive measures. Furthermore, in this context of a high number of individuals who already developed SARS-CoV-2 antibodies, the cost-effectiveness and risk-benefit of current vaccination strategies targeting vulnerable populations such as dialysis patients should be discussed.

## Data Availability

The data-sets used and/or analysed during the current study available from the author [Dr Moustapha Mbow, Immunology Lab, UCAD, Dakar, Senegal. Contact: moustapha.mbow@ucad.edu.sn] on reasonable request.
